# Chronic Orofacial Pain: Models, Mechanisms, and Genetic and Related Environmental Influences

**DOI:** 10.3390/ijms22137112

**Published:** 2021-07-01

**Authors:** Barry J. Sessle

**Affiliations:** 1 Faculty of Dentistry, University of Toronto, 124 Edward St, Toronto, ON M5G 1G6, Canada; barry.sessle@utoronto.ca; Tel.: +1-416-9463742; 2 Department of Physiology, Faculty of Medicine, University of Toronto, 124 Edward St, Toronto, ON M5G 1G6, Canada; 3 Centre for the Study of Pain, University of Toronto, 124 Edward St, Toronto, ON M5G 1G6, Canada

**Keywords:** orofacial pain, trigeminal, animal models, strain differences, environmental factors, genetic factors

## Abstract

Chronic orofacial pain conditions can be particularly difficult to diagnose and treat because of their complexity and limited understanding of the mechanisms underlying their aetiology and pathogenesis. Furthermore, there is considerable variability between individuals in their susceptibility to risk factors predisposing them to the development and maintenance of chronic pain as well as in their expression of chronic pain features such as allodynia, hyperalgesia and extraterritorial sensory spread. The variability suggests that genetic as well as environmental factors may contribute to the development and maintenance of chronic orofacial pain. This article reviews these features of chronic orofacial pain, and outlines findings from studies in animal models of the behavioural characteristics and underlying mechanisms related to the development and maintenance of chronic orofacial pain and trigeminal neuropathic pain in particular. The review also considers the role of environmental and especially genetic factors in these models, focussing on findings of differences between animal strains in the features and underlying mechanisms of chronic pain. These findings are not only relevant to understanding underlying mechanisms and the variability between patients in the development, expression and maintenance of chronic orofacial pain, but also underscore the importance for considering the strain of the animal to model and explore chronic orofacial pain processes.

## 1. Introduction

Orofacial pain conditions are very common and many that are chronic may be especially difficult to diagnose and treat because of their complexity and lack of clarity of the detailed mechanisms underlying their aetiology and pathogenesis [1,2,3,4,5,6]. Examples are temporomandibular disorders (TMDs) and several trigeminal neuropathic pain conditions. They typically display allodynia and hyperalgesia, but may be complicated by their expression of one or more comorbidities such as depression, anxiety, stress, sleep disturbances, and comorbid pain as well as extraterritorial sensory abnormalities and spread of sensitivity (ETSS) to widespread areas innervated by the trigeminal nerve or even to tissues supplied by spinal nerves (for review, see [2,4,7,8,9,10,11]). There can also be sex differences in some pain conditions as well as considerable variability between pain patients of the same or different racial groups in their expression of these features of chronic pain, treatment response, and susceptibility to risk factors that may predispose them to develop or maintain chronic pain [3,4,12,13,14,15,16]. These findings suggest that genetic as well as environmental factors may contribute to the development and maintenance of chronic pain and its expression in the orofacial region.

The variability in the expression of many of these chronic orofacial pain features, such as allodynia, hyperalgesia and ETSS, raises questions whether animal models of chronic orofacial pain display these features and whether there are differences in them between animal strains. These questions bear on the role of genetic and environmental influences in chronic orofacial pain. This article will review these features and also outline findings from studies of the spinal and trigeminal nociceptive systems in animal models that have shed some light on mechanisms underlying chronic orofacial pain and trigeminal neuropathic pain in particular. In addition, strain differences in these animal models will be described that provide insights into the role of genetic as well as environmental factors that are relevant to the variability between patients in the development, maintenance, and features of chronic orofacial pain and in responses to its treatment.

## 2. Chronic Orofacial Pain States: Features, Animal Models and Mechanisms

### 2.1. Features of Chronic Orofacial Pain States

The orofacial region is a common site of chronic pain conditions, some of which are unique to this part of the body (for review, see [6,17,18]). They range from those for which there is a clear aetiology (e.g., trigeminal postherpetic neuralgia, post-traumatic trigeminal neuropathic pain [PTNP]) to those that may be idiopathic with no clearly recognizable cause (e.g., burning mouth syndrome [BMS]; persistent idiopathic facial pain [PIFP]; persistent idiopathic dentoalveolar pain [PIDP] to others that are expressed as part of a recognized chronic disorder or disease (e.g., arthritis, cancer, diabetes, HIV/AIDS). Moreover, approximately 20% of acute pains can transition into a chronic pain state if the acute condition is not managed effectively in a timely and appropriate manner [19,20,21,22,23]. The recently published comprehensive classification of all types of orofacial pain conditions by an international multidisciplinary collaboration can be consulted for an up-to-date detailed outline of these conditions [18].

As noted above, diagnosis and treatment of chronic orofacial pain states can be complicated by the lack of clarity of underlying mechanisms and comorbid features. Furthermore, the presence of chronic pain in the orofacial region can in itself add an extra layer of complexity because of the special meaning of the face and mouth to humans for communication from one person to another through speaking and expression of “feelings” and for carrying out important functions related to taste, smell, chewing, swallowing, and associated sensorimotor functions [17,24,25]. Another complication stems from the very nature of pain, since pain has a multidimensional character. Pain encompasses sensory-discriminative, affective-motivational, and cognitive-evaluative dimensions, consistent with the biopsychosocial model of pain [26,27]. These dimensions are especially apparent in chronic pain states, and may further complicate their diagnosis and management.

What is particularly noteworthy from the perspective of the focus of this review are features that are hallmarks of most chronic orofacial pain conditions; these include (i) the characteristics of the pain expressed in the orofacial region innervated by the trigeminal nerve, (ii) the presence of comorbid pain or spread of sensitivity within or even beyond the trigeminal region (i.e., ETSS), and (iii) the variability between individuals in the development of a chronic pain condition following an injury, in the time period over which the pain is maintained, in the characteristics of that pain condition, and in the response to treatment (e.g., [28,29,30,31,32]; for review, see [2,4,6,10,16,33,34,35]). The following will primarily focus on trigeminal neuropathic pain conditions to consider each of these three features in turn.

Firstly, the characteristics of most chronic orofacial pain states have many similarities with characteristics such as allodynia and hyperalgesia that in general are commonly manifested in chronic pain states elsewhere in the body. Allodynia refers to pain evoked by a stimulus (e.g., tactile; cool air) that does not normally produce pain or tissue damage. It is a common feature of most chronic orofacial pain conditions, such as trigeminal neuralgia, PTNP, and TMDs, although allodynia is also commonly experienced after an acute injury or inflammation when even a light mechanical (tactile) stimulus may evoke pain from the affected tissue. On the other hand, hyperalgesia is a term where an exaggerated pain sensitivity is produced by a noxious stimulus; it is also a feature of several chronic or acute orofacial pain conditions. In some cases, the pain experienced by the patient may be instead or as well reflected in a “spontaneous” pain that may be ongoing and/or paroxysmal, even occurring in some conditions without the presence of any clear stimulus; ongoing pain is not uncommon, for example in BMS, PTNP and a recently classified condition known as Constant Unilateral Facial Pain with Additional Attacks (CUFPA), and paroxysmal pain is a common feature of CUPFA, trigeminal neuralgia, and PTNP [6,18].

Secondly, like chronic pain conditions in other parts of the body, it is also common for many chronic orofacial pain states to be associated with comorbid negative psychosocial features and disturbances as well as overlapping pain elsewhere in the body [6,7,8,11,27]. For example, TMD patients often have comorbid migraine headache, irritable bowel, or fibromyalgia, and commonly express anxiety, stress, depression and sleep disturbances; most chronic orofacial neuropathic pain conditions are also usually accompanied by these psychosocial features and disturbances. It is also not uncommon in some chronic orofacial pain states for the pain to be diffuse (e.g., BMS, PIFP) or for the pain or sensory abnormality in one part of the orofacial region innervated for instance by one trigeminal nerve division to spread or be referred to other parts of the face or mouth supplied by one or two other divisions of the trigeminal nerve or even spread to extra-trigeminal regions (i.e., ETSS) [6,35,36,37]. One example is PTNP and its diagnostic contrast with some other trigeminal neuropathic pain conditions. PTNP is a neuropathic pain condition with a definitive cause, i.e., an injury in the form of a nerve trauma or disease involving branches of the trigeminal nerve. One of its diagnostic features is that the pain and any associated somatosensory disturbances are confined to the trigeminal division of the injury or disease [6,18,38]. If, however, the pain condition displays ETSS, crossing boundaries beyond what appear to be the relevant trigeminal nerve branches, then an idiopathic pain such as PIFP may be the correct diagnosis [6,18,37]. Another example is TMDs where, in addition to the comorbid conditions associated with them (see above), pain may sometimes be experienced not only in the temporomandibular area but also in parts of the neck supplied by cervical spinal nerves. Furthermore, TMD patients and patients with some forms of headache may also display ETSS reflected in abnormal sensitivity that can be detected with quantitative sensory testing (QST) as altered sensory thresholds in parts of the body such as the hand, well beyond trigeminal neuroanatomical boundaries and the major orofacial source of the pain (e.g., [13,30,31,32,36,39,40,41,42]). It should also be noted that these features are not limited to orofacial pain conditions since there are clinical reports suggesting the reverse situation, i.e., that pain in tissues supplied by spinal nerves can be associated with ETSS to the orofacial region (e.g., [43,44,45]). Furthermore, ETSS to contralateral tissues (“mirror-image pain”) can also occur following unilateral spinal nerve injury (e.g., [46,47]).

The third feature to consider about chronic orofacial pains is another one that they share with many chronic pains occurring in other parts of the body. This is the considerable variability between individuals and even between racial groups in (i) the signs and symptoms of the pain, (ii) the occurrence and time course of comorbidities and ETSS, and (iii) the development of a chronic pain state in the first place, and its time course [3,8,15,28,29,42,48,49,50,51,52]. Variability is especially evident in patients who have experienced trigeminal nerve trauma through injury or disease. Like patients with neuropathic pain following spinal nerve trauma, those with a trigeminal neuropathic pain condition are heterogeneous in the aetiology, pathogenesis and clinical features of the condition. It is not uncommon for injury to occur to lingual nerve, inferior alveolar nerve (IAN) or infraorbital nerve (ION) branches of the trigeminal nerve because of an accidental traumatic event (e.g., motor vehicle accident, sporting injury). These nerves are also commonly traumatised as a result of rehabilitative or surgical procedures such as tooth extraction, endodontic therapy, local anaesthetic nerve block, dental implant placement, or orthognathic surgery. Yet there is great variability in the occurrence and time course of chronic neuropathic pain following these injuries; for example, while each of these dental procedures has no persistent sequelae in most patients, but can produce chronic neuropathic pain (e.g., PTNP) in anywhere from 3 to 15% of patients receiving these procedures [3,6,53,54,55]. Not only is there variability in the incidence of chronic neuropathic pain following a trigeminal nerve injury, but there is also great variability between individuals and different racial groups in the signs and symptoms expressed (e.g., levels of allodynia, hyperalgesia, ETSS, psychosocial parameters) [3,4,15,16,23,51,52,54,55,56]. On the surface of it, such variability may not seem surprising since different levels of injury may be produced by the same type of accident or therapeutic procedure. Nonetheless, variability is still a feature despite the apparently similar extent of the nerve and tissue trauma caused by the accidental injury or by the surgical or local anaesthetic approach carried out under standard operating procedures, and the intensity of the pain expressed cannot be readily predicted from the severity of the injury.

**Figure 1:**
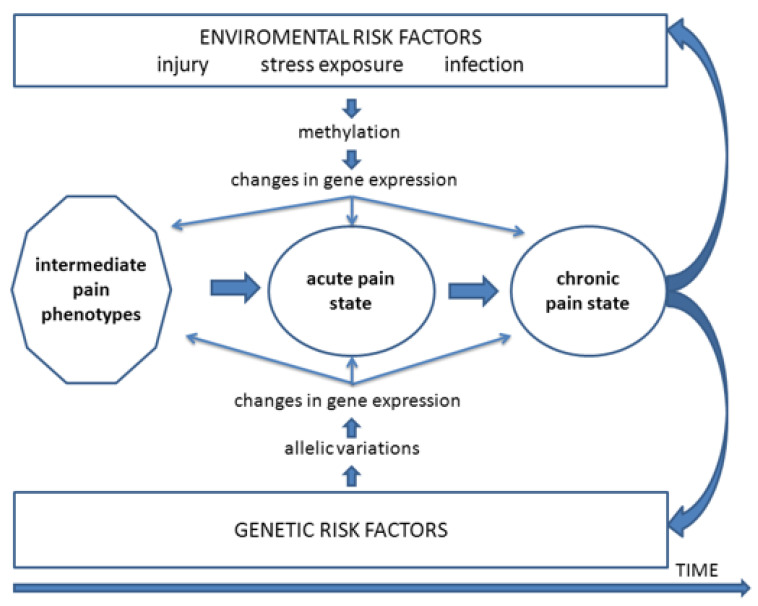
Mechanistic outline of processes by which genetic and environmental risk factors may influence orofacial pain. These include influences on intermediate pain phenotypes and the transition from an acute pain state to a chronic pain state that may further exacerbate these risk factors. From Meloto et al. [15].

It is important to recognise that for those chronic neuropathic pain states that occur following injury to branches of the trigeminal nerve, the nerve injury itself represents an “environmental” factor that is the inciting event producing nociceptive afferent inputs into the central nervous system (CNS) that are critical in the initiation of the pain state. In addition, the variability in expression of pain and its features between individuals of the same or different racial background suggests the involvement also of genetic or epigenetic factors in the biological processes or psychosocial factors bearing on chronic pain, thereby influencing pain expression and predisposing patients to the initiation and/or the maintenance of a chronic neuropathic pain state following trigeminal nerve injury. Figure 1 shows examples of how genetic and environmental risk factors may influence orofacial pain and the transition from an acute pain state to a chronic pain state. The role of genetic or epigenetic influences is also consistent with the well-documented differences in pain sensitivity between healthy individuals of the same or different racial groups as well as between males and females (e.g., [12,14,33,57,58]; for review, see [3,4,9,16,54]). The variability in pain sensitivity as well as in the development and expression of chronic orofacial pain raises the question whether in animal models of orofacial neuropathic pain there are differences between strains of animals in the expression of nociceptive behaviour and underlying mechanisms such as trigeminal central sensitization. The answer to this question bears on the role in orofacial pain of environmental influences and of genetic influences in particular. To address this question, the following considers firstly findings from animal models of neuropathic pain of the behavioural characteristics and underlying mechanisms related to the development and maintenance of chronic orofacial neuropathic pain following injury to the trigeminal nerve. Consideration is then given to the role of environmental and especially genetic factors in these models, focusing on findings of differences between strains of animals in the expression of features of a chronic pain state.

### 2.2. Animal Models

The use of animal models of chronic orofacial pain has produced findings underpinning the general view that the peripheral changes (e.g., peripheral sensitization, ectopic afferent inputs) and CNS changes (e.g., central sensitization) that can result from a peripheral injury or inflammation are involved to varying degrees in orofacial pain states, including the development of neuropathic pain conditions expressed in the orofacial region such as PTTN, BMS, trigeminal neuralgia, and trigeminal postherpetic neuralgia (for review, see [25,35,59,60,61,62,63,64,65,66]). Several models of orofacial neuropathic pain have been developed to document these changes, but before considering them, some general features about these models and mechanisms should be noted. Firstly, although it is clear that trigeminal nerve injury produces behavioural and associated CNS changes that generally are analogous to those produced by spinal nerve injury (for review, see [37,61,64]), the orofacial region has some unique tissues and pain syndromes (e.g., trigeminal neuralgia, BMS, PDIP), and there are several notable differences in the functional and morphological features of the trigeminal and spinal systems and in underlying processes related to pain (e.g., [63,67,68]; and see below). As a consequence, it cannot be assumed that the effects of trigeminal nerve injury are the same as those occurring after spinal nerve injury. Secondly, while several animal models of neuropathic pain in the trigeminal system have been developed, definitive models of most orofacial neuropathic pain conditions are lacking, and most of those that have been developed to mimick a specific clinical pain state (e.g., trigeminal neuralgia) have been criticized since their phenotypic features do not necessarily match the human clinical pain syndrome that they are supposed to be modelling (e.g., [38,59,68,69,70]). Thirdly, the point above about translatability of animal models of orofacial pain to clinical pain states brings up, in a broader context, the limitations of animal models of pain and what alternatives there may be to help elucidate pain mechanisms (for review, see [71,72,73,74]). One long-standing limitation is the translatability to the clinic of pre-clinical findings obtained in animal models of analgesic therapies. The mixed results in the translation of analgesic drug development from findings in animal pre-clinical models to human chronic pain conditions is well known, due in part to species differences and to the inappropriateness of the model selected to mimick a clinical pain condition as noted above. However, other factors have also been identified, such as the difficulty in defining the time-points of acute to chronic pain transition in animal models relative to that in humans (where 3 months is usually considered the time-point for a pain state to be considered chronic; see [18,75]), as well as the possibility of use-dependent alterations in analgesic drug sensitivity and time-dependent changes in nociceptive processes in vivo in specific animal models (e.g., [74,76]). However, challenges such as these which confront the use of animal models of pain are not unique to this research area since they also apply to numerous therapeutic fields. It is notable that there have been concerted efforts in recent decades to limit the use of in vivo approaches or to reduce the number of in vivo animal experiments required to address a particular question about pain mechanisms (for review, see [71,72,73,74,76]); examples include immortalised cell lines, explant cultures, in vitro electrophysiological recording and immunohistochemical labelling, and psychophysical and neuroimaging studies in humans. Additionally, there are other emerging approaches that will help in minimising or even avoiding the use of animals in some pain research areas, such as those approaches using tissues on chips, human-derived three-dimensional tissue models, artificial intelligence and computer modelling. Nonetheless, despite the challenges and limitations, animal models of pain, complemented by these other approaches, will undoubtedly continue to be essential for improving understanding of mechanisms of pain and its control.

The animal models of orofacial neuropathic pain that have been developed over the past several decades include those involving lesions made within peripheral or CNS components of the trigeminal somatosensory system. They range from a few models where surgical or chemical disruption is produced in CNS components of the trigeminal nociceptive system such as the trigeminal brainstem sensory nuclear complex (TBSNC) to a much larger group of models where trigeminal nerve branches or trigeminal ganglion are manipulated by procedures such as transection or constriction of the IAN or ION or compression of the trigeminal ganglion or sensory root (for review, see [17,35,38,59,69]). The following focusses on findings from this larger group of models, providing an overview of the alterations in peripheral and central components of the trigeminal nociceptive system and the associated behavioural changes that occur following trigeminal nerve injury. The overview cites several of the many research reports related to this topic to exemplify the progress in using animal models to address these effects of trigeminal nerve injury; for more extensive reviews, see [35,59,62,63,66].

### 2.3. Cellular Mechanisms and Associated Behaviour

**Figure 2:**
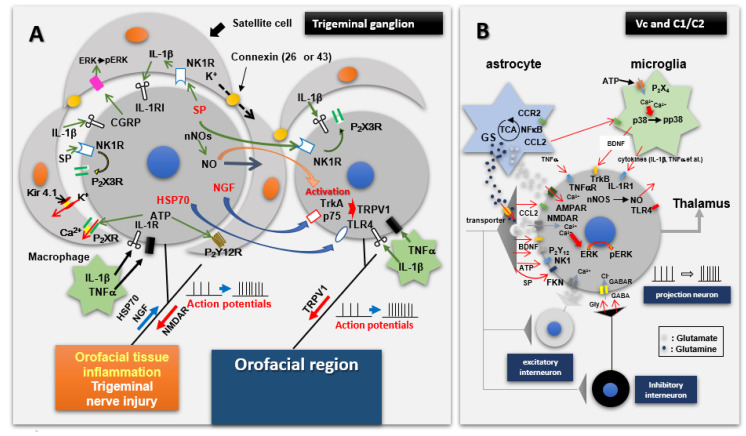
Mechanisms involved in initiation and transmission of nociceptive afferent information from orofacial tissues. (**A**) shows that trigeminal nerve injury or orofacial inflammation can induce hyperexcitability of trigeminal primary afferent neurons, satellite glial cell activation and macrophage accumulation. The hyperactivated trigeminal ganglion (TG) neurons as well as the satellite glial cells and macrophages communicate with each other through a variety of mediators, receptor mechanisms and signalling processes, many of which are shown here. Through this communication, further enhancement of the excitability of TG afferent neurons may occur, as manifested in their hyperexcitable input into components of the trigeminal brainstem sensory nuclear complex, in particular the medullary dorsal horn (also known as trigeminal subnucleus caudalis [Vc]), as well as the upper cervical spinal cord (C1/C2). (**B**) shows input and output features of nociceptive neurons in the medullary dorsal horn and C1/C2 under normal conditions as well as pathological conditions such as trigeminal nerve injury or orofacial inflammation which produces the hyperexcitable nociceptive afferent input shown in A. This input releases mediators which cause the neurons to become hyperactive, resulting in activation of microglia and astrocytes. Neuron–glial cell communication via various molecules, several of which are shown here, is important in fostering the hyperexcitable state of the neurons, i.e., central sensitization. From Iwata and Sessle [25].

Injury to the trigeminal nerve produces alterations in trigeminal primary afferents that can be manifested in a variety of changes, many of which can also result from other types of injury, orofacial inflammation, or injection of algesic chemicals into orofacial tissues (for review, see [35,38,59,61,63,66]). Most notable is peripheral sensitization which is a hyperexcitable state of the afferents that is reflected in abnormally increased evoked responses to noxious stimuli, lowered activation threshold, spontaneous activity that may include abnormal bursting discharges, and spread of sensitivity to adjacent afferents. These features may contribute to spontaneous pain and to the increased sensitivity expressed for example as allodynia and hyperalgesia when an injured or inflamed peripheral tissue is contacted. Peripheral sensitization can be accounted for by the release of proinflammatory mediators (e.g., TNF-alpha, IL1-beta, IL6) as well as changes in several neurochemicals and molecular and receptor mechanisms such as those involving transient receptor potential receptors (e.g., TRPV1), nerve growth factor receptors (e.g., NGF), purinergic receptors (e.g., P2X, P2Y), neurokinin receptors (e.g., substance P, calcitonin gene related peptide [CGRP]), cannabinoid receptors (e.g., CB2) and glutamatergic receptors (e.g., NMDA, metabotropic) as well as calcium, sodium, and potassium channels. Some of these processes display sex differences, and it is also notable that the processes may occur in or around the peripheral endings of the primary afferents as well as or instead in their cell bodies in the trigeminal ganglion (see Figure 2). The changes in the ganglion include upregulation of ganglion satellite glial cells, and up or down regulation of signalling processes and proinflammatory agents and other chemical mediators that are released in the ganglion from satellite glial cells, immune cells, macrophages and neurons. By these mechanisms, these neurons and cells can communicate with each other and contribute to the hyperexcitability of the afferents and their inputs into the TBSNC. It is notable that some of the changes may be specific to the trigeminal system since there are some differences in these processes between the trigeminal and spinal systems [63,68,77,78,79,80,81]. For example, trigeminal nerve injury leads to some genes (e.g., metabotropic glutamate receptor 5, CB receptor 2, TNF-alpha, 5-hydroxytryptamine receptor 1a) being upregulated in the trigeminal ganglion at certain times after trigeminal nerve injury whereas these genes in the dorsal root ganglia of the spinal system are typically downregulated. In addition, spinal nerve injury can result in sympathetic nerve sprouting in the dorsal root ganglia, but trigeminal nerve injury has been reported to produce no sprouting of sympathetic nerves in the trigeminal ganglion. Furthermore, hyperexcitability of trigeminal afferents may be less than that of comparable spinal afferents following injury, respectively, of trigeminal nerves vs. spinal nerves.

Despite these specific differences, it is noteworthy that in both trigeminal and spinal systems, the changes in the excitability of afferents following nerve injury (or inflammation) may not be limited to the damaged afferents. Through the action of gap junctions, satellite glial cells and paracrine processes involving chemical mediators released in the ganglion and through analogous paracrine processes in the vicinity of the peripherally damaged tissue, undamaged afferents may also be altered (for review, see [35,52,59,82,83,84,85,86]). Changes may also occur in the peripheral innervation patterns of afferents spared from the nerve injury. Some of these intact peripheral afferents may continue to innervate their original receptive fields, but some may sprout locally to innervate any receptive fields left vacant by injured afferents cut off from their peripheral endings. Consequently, these intact afferents feed the TBSNC with abnormal stimulus-evoked sensory inputs which drive trigeminal nociceptive pathways that are normally somatotopically aligned with the uninjured afferent nerve fibres. As a result, an increased pain sensitivity reflected in extraterritorial secondary hyperalgesia may occur. Another contributing factor to such extraterritorial afferent inputs into the CNS is neurone–glial cell communication, and possibly direct communication between neurons, in the trigeminal ganglion. The interactions between satellite glia cells and neurons involve the release of chemical mediators and changes in signalling mechanisms that can result in spread of hyperexcitability throughout the different trigeminal divisions of the trigeminal ganglion (see Figure 2). For example, through such processes, injury to primary afferents in the ION, which is a branch of the second (maxillary) division of the trigeminal nerve, may increase the excitability of not only primary afferent neurons in the maxillary division of the trigeminal ganglion but also neurons in the other two trigeminal divisions of the ganglion. This process represents another peripheral mechanism by which ectopic trigeminal afferent inputs beyond the territory of the affected afferents may access the TBSNC and lead to the development of extraterritorial secondary hyperalgesia, as Figure 2 illustrates. Collateral sprouting of the central endings of the damaged primary afferents that engages neurons in widespread areas of the TBSNC may further add to the extraterritorial nature of the afferent inputs following trigeminal nerve injury.

**Figure 3:**
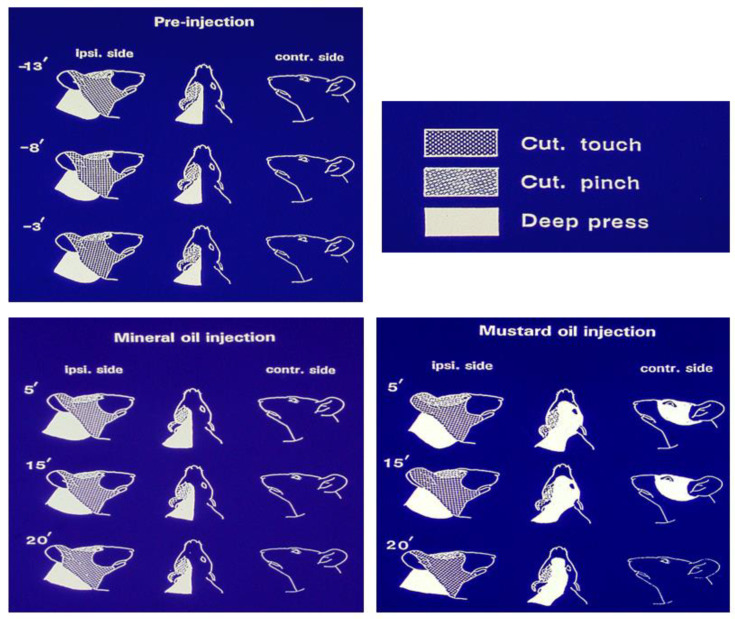
Features of a nociceptive neuron recorded in the rat medullary dorsal horn that exhibited extensive mechanoreceptive field expansion as a reflection of trigeminal central sensitization induced by intramuscular injection of the inflammatory irritant mustard oil. The neuron was a wide dynamic range neuron since it could be activated by tactile stimulation of the cutaneous periorbital area as well as by pinch stimulation of the posterior facial skin and by heavy pressure applied to posterior cranial and cervical tissues. The figurines show that in the control condition (pre-injection), the mechanoreceptive field was limited to the ipsilateral side and was stable for several minutes, and also did not change over a 20-min period following injection of vehicle control (mineral oil) into the tongue musculature. However, note that within 5 min after the injection of the inflammatory irritant mustard oil into the tongue, the mechanoreceptive field had expanded to encompass the ipsilateral ear as well as contralateral facial and cervical areas. The expanded mechanoreceptive field was maintained for at least another 10 min before returning towards pre-injection features by 20 min. Based on data from Yu et al. [89].

In the case of nociceptive transmission from the central endings of the nociceptive primary afferents to neurons in the TBSNC, this process may take place in the rostral components of the TBSNC (e.g., subnucleus oralis) but is especially evident in its more caudal components, namely the medullary dorsal horn (MDH, also known as trigeminal subnucleus caudalis) and the caudalis–interpolaris transition zone, as well as in the upper cervical dorsal horns. The nociceptive transmission process involves the release of several neurochemicals from the central endings of the primary afferents, as illustrated in Figure 2 (for review, see [35,38,59,61,65,66,87]). In the situation where there are abnormal hyperexcitable or ectopic afferent inputs (see above), neuroplastic changes may be produced in the TBSNC that are expressed as an increased excitability of nociceptive neurons in the TBSNC. Several mediators, receptors and signalling processes have been identified as crucially involved in the production of the increased excitability of the nociceptive neurons. These include glutamatergic, neurokinin (e.g., substance P; CGRP) and purinergic (e.g., ATP) mediators released from the primary afferents, intracellular signalling processes such as nitric oxide and pERK, as well as mediators such as cytokines released from other cells (e.g., glia) within the TBSNC, as illustrated in Figure 2. The neuronal hyperexcitability reflects a central sensitization which in the TBSNC has been documented by immunohistochemical methodologies (e.g., increased expression of p-ERK or the proto-oncogene c-fos) and especially defined by electrophysiological recordings in the MDH and adjacent regions. The neuroplastic changes related to central sensitization are manifested as electrophysiological alterations in the receptive field and response properties of the nociceptive neurons that are of two main types: nociceptive-specific neurons which are activated only by noxious stimuli applied to their receptive field, and wide dynamic range neurons which are activated by non-noxious (e.g., tactile) as well as noxious stimuli. The neuroplastic changes include a decrease in activation threshold to stimuli applied to the neuronal receptive field, as well as increases in responses to noxious stimuli, receptive field size, and spontaneous activity (for review, see [25,35,59,62,65,66,84,87,88]. Examples of these neuronal parameters of trigeminal central sensitization are shown in Figure 3 and also further below in Section 3.

The neuronal parameters of central sensitization, along with some features of peripheral sensitization reflecting hyperexcitability of afferent inputs, are consistent with the sensory experiences of human subjects under acute or chronic experimental orofacial pain conditions [2,4,10,54]. They contribute to the allodynia, hyperalgesia, pain spread and spontaneous pain that are typical clinical features of many types of chronic pain, neuropathic or otherwise. Indeed, trigeminal central sensitization is also apparent in animal models of orofacial inflammatory or surgically induced pain, as exemplified by the expanded receptive fields of MDH nociceptive neurons that may include contralateral or cervical areas. An example is shown in Figure 3 where compared with control values, mechanoreceptive fields expanded 143%, 187% and 140%, respectively, for the pinch, tactile and deep pressure components of the receptive fields that encompassed contralateral facial and cervical areas at 5 min after the intramuscular injection of the inflammatory irritant mustard oil [89]. Findings such as these in a variety of pain models have resulted in central sensitization being invoked as an integral element underlying acute and chronic pain conditions. For example, central sensitization is generally considered a critical factor underlying TMD pain, the increased sensitivity to pressure stimulation of the temporomandibular area in TMD patients, and to contribute to its comorbid or overlapping pains, irrespective of whether they are considered discrete entities or components of the one single syndrome (e.g., [2,5,6,28,31,65,90,91]).

The central sensitization in the TBSNC that involves alterations in chemical mediators and molecular processes underlying trigeminal nociceptive transmission in the TBSNC is a reflection of glioplasticity as well as neuroplasticity (Figure 2). The important role of glia (astrocytes and microglia) in the development and maintenance of trigeminal central sensitization is exemplified in animal models of trigeminal neuropathic pain by immunohistochemical changes, reflecting their upregulation in the MDH and adjacent regions after trigeminal nerve injury and by evidence that trigeminal central sensitization and accompanying nociceptive behaviour can be overcome by administration of glial cell inhibitors (for review, see [35,59,62,66,92]). This is consistent with some, although not all, clinical studies of the efficacy of agents inhibiting glial cell processes in craniofacial pain conditions [85,93,94,95]. It is also noteworthy that the neuroplastic changes underlying central sensitization are not limited to the nociceptive neurons in the MDH but can also occur in the upper cervical spinal dorsal horn and other components of the TBSNC and may be accompanied by enhanced excitability of motoneurons supplying jaw, tongue and cervical muscles (for review, see [24,35,59,62,65,66]). Although sex differences occur in several chronic orofacial pain states (see Section 2.1 above), only limited study has been made in animal models, but there is evidence that sex differences do exist in the neuronal or glial cell processes contributing to trigeminal nociceptive transmission and central sensitization in the TBSNC or in other parts of the trigeminal system [61,96,97].

Limited study has also been made of neuroplastic or glioplastic changes and central sensitization occurring in higher brain centres in the orofacial models, but neuroplasticity, central sensitization and in some cases glioplasticity have been documented in the somatosensory thalamus and other CNS areas involved in transmission or modulation of trigeminal nociceptive information following orofacial injury or inflammation (e.g., [98,99,100,101,102,103]). Such findings are consistent with neuroimaging observations in humans of activation or modulation in these higher levels of the CNS in chronic orofacial pain states [104,105,106]. Furthermore, manipulations of the MDH such as ibotenic acid lesioning or inhibition of the processes underlying central sensitization (e.g., i.t. application of a glutamatergic, cytokine, astroglial or microglial inhibitor) have revealed that the behavioural hypersensitivity and the central sensitization occurring in other components of the trigeminal nociceptive pathways in the CNS depend on the functional integrity of MDH or its transitional region with subnucleus interpolaris (e.g., [107,108,109,110]; for review, see [35,59,62,65,66,85,92]). The net result of the changes in the intrinsic modulatory circuits following nerve injury or inflammation is an enhancement of facilitatory influences and a decrease in inhibitory influences that reinforce the hyperexcitability of the nociceptive neurons initiated by the abnormal afferent inputs to the neurons that were evoked by the injury or inflammation. The neuronal hyperexcitability may not be readily reversible, and as a result the central sensitization may become sustained, resulting in a chronic state of nociceptive neuronal hyperexcitability. It should, however, be noted that the changes in the intrinsic modulatory circuits are not the only factor involved in reinforcing neuronal hyperexcitability and in contributing to the transition from an acute to a chronic pain state. The transition process has been a particular focus of pain research in recent decades, and the complexity of the mechanisms involved is underscored by the several neural circuits and non-neural as well as neural processes that have been implicated. For example, there is evidence that the mechanisms include neuroplastic and glioplastic processes underlying peripheral and central sensitization, genetic priming, and changes in descending pain-modulatory pathways such as those involving the rostral ventromedial medulla and corticolimbic circuits, in association with the interplay of a variety of demographic, psychosocial, and injury-related risk and protective factors (e.g., [20,22,34]).

In the animal models of orofacial neuropathic pain, the animal’s behaviour typically shows features that are generally reflective of the neuronal changes in excitability that have been outlined above in terms of peripheral sensitization and central sensitization. The features are indicative of the clinical characteristics of most chronic pain states, namely allodynia, hyperalgesia, pain spread, and spontaneous pain (for review, see [35,59,62]). Typically, the animal’s behaviour shows mechanical and/or thermal hypersensitivity to peripheral stimuli, and spontaneous pain-like behaviour; other behavioural changes indicative of pain may also be evident, such as facial grimacing and other altered sensorimotor behaviours (e.g., reduced chewing or locomotion). Another notable feature in animal models of the nociceptive behaviour following unilateral nerve injury is the appearance of hypersensitivity beyond the peripheral innervation territory of the injured nerve, consistent with reports of ETSS not only clinically in trigeminal neuropathic pain states but also in experimental pain studies in humans (e.g., [13,14,30,31,32,36,39,40,41,42]; for review, see [2,4,6,10,16,35]).

While peripheral mechanisms such as the ectopic primary afferent inputs to the TBSNC (see above, and Figure 2) may contribute to ETSS within ipsilateral orofacial tissues following unilateral injury, central mechanisms are considered to underlie ETSS extending to contralateral orofacial tissues or more widespread areas. Some characteristics of central sensitization, notably the functional changes reflecting neuronal hyperexcitability and the accompanying immunohistochemical alterations expressed in the nociceptive neurons and glial cells (see above), can explain these features of ETSS. For example, unilateral injury of trigeminal nerve branches in the ION or IAN can result in neuropathic pain-like behaviours which include ETSS that extends beyond the ipsilateral orofacial tissues innervated by the nerve that has been injured. This behavioural change is accompanied by immunohistological changes in neurons and glia that are widespread in the MDH, extending beyond the MDH region normally representing the territory of the injured nerve. Likewise, the electrophysiologically defined alterations reflected in central sensitization of both wide dynamic range and nociceptive-specific neurons include neuronal receptive fields that have expanded beyond the innervation territory of the injured nerve and may encompass afferent inputs from extraterritorial trigeminal divisions (for review, see [35,59,62,66,92]). This change in the spatial coding features of centrally sensitized nociceptive neurons can explain the common characteristic of the accompanying nociceptive behaviour that is expressed as ETSS following a unilateral trigeminal nerve injury, i.e., mechanical and/or thermal hypersensitivity manifested extraterritorially in the ipsilateral orofacial region beyond the trigeminal division of the injured nerve as well as in contralateral orofacial areas. These immunohistochemical, electrophysiological and behavioural expressions of ETSS are not limited to animal models of trigeminal neuropathic pain but are also apparent in models of craniofacial inflammatory or surgically induced pain (e.g., [89,109,110,111,112,113,114,115,116,117]; for review, see [61,62,65,66,88]). Reference again to Figure 3 illustrates this feature for MDH nociceptive neurons with expanded receptive fields that even include tissues (cervical) supplied by spinal nerves.

**Figure 4:**
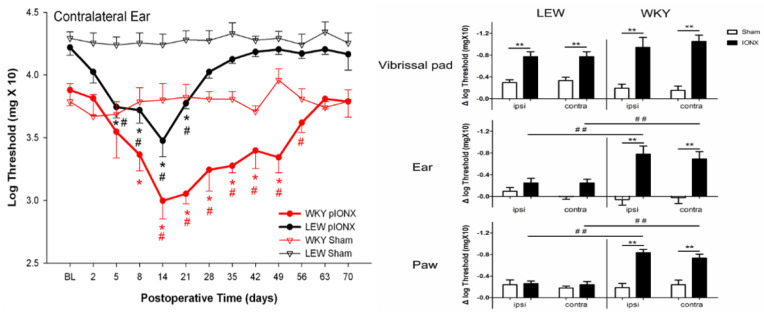
Infraorbital nerve injury in the rat induces hypersensitivity that spreads extraterritorially and is genetically dependent. In adult male WKY and LEW rats housed under similar environmental conditions, head withdrawal thresholds to mechanical stimulation of the facial vibrissal pads, ears and hindpaws were assessed bilaterally with von Frey monofilaments before (baseline [BL]) and after receiving unilateral transection (IONX) of the medial branch of the infraorbital nerve (that supplies medial aspect of upper lip, vibrissal pad and anterior teeth) or sham operation (*n* = 10/group). On the left is an example of the time course of the effect of the partial IONX (in comparison to sham) in inducing extraterritorial spread of sensitivity (ETSS) as reflected in a significant decrease in the withdrawal threshold to mechanical stimulation of the contralateral ear (2-way ANOVA, *p* < 0.05). This reflection of IONX-induced nociceptive behaviour occurred in both strains but was significantly greater and longer lasting in the WKY rats. Additional evidence of ETSS is illustrated on the right where WKY rats showed, at 3–5 weeks post-IONX, significantly (2-way ANOVA, *p* < 0.05) decreased mechanical withdrawal thresholds not only in the contralateral ear but also in in the lateral part of the partially denervated ipsilateral vibrissal pad, and ear and hindpaw. LEW rats also displayed significant ETSS but only for the vibrissal pad; their levels of mechanical hypersensitivity in the ear and hindpaw were significantly lower (*t*-test, *p* < 0.05) than those in WKY rats. They also showed significantly lower levels of heat hypersensitivity in these sites that were tested by applying noxious infrared laser heat stimuli (200 msec, 18–25 Amp, 2 mm in diameter diode) to these sites (not shown). * *p* < 0.05 comparison to baseline, ** *p* < 0.05 and ^#^ *p* < 0.05, ^##^ *p* < 0.05 comparison between groups. Based on data from Wang et al. [120].

**Figure 5:**
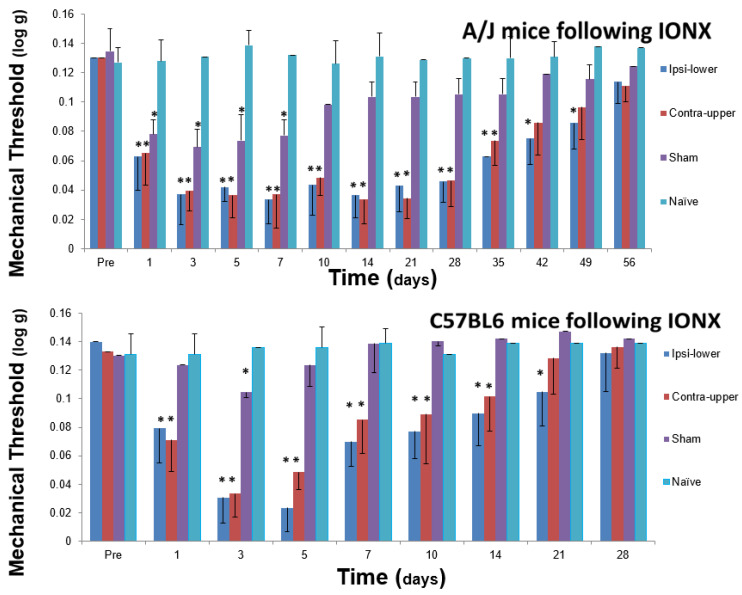
Infraorbital nerve injury in the mouse induces facial mechanical hypersensitivity that is genetically dependent. A/J and C57BL/6J male mice aged 8–12 weeks were maintained under similar environmental conditions, and were tested with von Frey monofilaments for facial mechanical sensitivity before and after unilateral transection (IONX) of the medial branch of the infraorbital nerve (that supplies medial aspect of upper lip, vibrissal pad and anterior teeth) or sham operation; naïve mice were also tested (*n* = 11/group). Baseline values of facial mechanical sensitivity as reflected in head withdrawal threshold were comparable in A/J and C57BL/6J mice, and following IONX both strains showed evidence of ETSS as reflected in hypersensitivity in the ipsilateral lower lip and contralateral upper lip when compared with the sensitivity in naïve and sham animals. However, the time course of the IONX-induced facial hypersensitivity was significantly different (2 way ANOVA, *p* < 0.05) between the two strains of mice: hypersensitivity in both A/J and C57BL/6J mice started around post-operative day 1, but in the A/J mice reached its peak throughout post-operative days 3–21 and lasted up to day 49, whereas in the C57BL/6J mice, hypersensitivity had a significantly shorter peak duration. Moreover, hypersensitivity lasted up to days 35 (contralateral upper lip) and 49 (ipsilateral lower lip) in A/J mice but only to days 14 and 21, respectively, in C57BL/6J mice. Sham animals (compared to naïve animals) showed a brief hypersensitivity that also was significantly longer in the A/J mice. * *p* < 0.05. Based on data from Varathan et al. [121] and Cherkas et al. [168].

The extension of ETSS to involve tissues innervated by spinal nerves also may occur in behavioural models of trigeminal neuropathic pain where the ETSS reflected in mechanical or heat hypersensitivity in widespread orofacial tissues can extend to extra-trigeminal regions supplied by spinal nerves [118,119,120,121]. Figure 4 and Figure 5 give examples in rats and mice, respectively, showing that unilateral transection of the medial branch of the ION supplying the medial upper lip (including vibrissal pad) and anterior teeth induces hypersensitivity not only in the ipsilateral face but also ETSS lasting several weeks in extraterritorial trigeminal areas (e.g., contralateral ear and upper lip, ipsilateral lower lip) and even in non-trigeminal regions such as the hindpaw (the differences in hypersensitivity between strains shown in these figures will be discussed in the section below). It should also be noted that the reverse situation can apply, whereby facial hypersensitivity may occur in animal pain models involving spinal nerve injury. For example, unilateral injury to cervical nerves or inflammation of tissues innervated by spinal nerves can produce bilateral facial hypersensitivity and trigeminal central sensitization associated with extraterritorially expanded neuronal receptive fields (e.g., [110,116,122]), consistent with the clinical reports noted earlier that nociceptive inputs into the CNS from tissues supplied by spinal nerves can induce ETSS to the trigeminal system. Such findings are also consistent with findings of ETSS in spinal neuropathic pain models showing features such as alterations to the nociceptive tail flick after cervical rhizotomies, spread of sensitivity ipsilaterally well beyond the boundaries of the injured nerve, contralateral allodynia and hyperalgesia, and expanded neuronal receptive fields in the spinal dorsal horn after a unilateral partial spinal nerve lesion (e.g., [47,123,124,125,126,127,128,129,130]).

The foregoing outline of features of ETSS between trigeminal and spinal nociceptive systems brings up another notable point about central sensitization and the accompanying behavioural changes, and this is their relevance to the comorbid pains and ETSS that, as noted earlier, may occur in different body regions in some chronic orofacial pain conditions. These clinical features very likely involve neuroplasticity and glioplasticity expressed as central sensitization occurring concomitantly in parts of the CNS involved in processing or modulating nociceptive information related to each of these different regions. There are documented pathways from the TBSNC and other brainstem areas (e.g., rostral ventromedial medulla, reticular formation) that receive trigeminal nociceptive inputs and send modulatory influences to areas of the spinal cord (e.g., spinal dorsal horn) involved in spinal nociceptive transmission and that could explain how centrally sensitized neurons of the TBSNC may impact spinal nociceptive circuits and related behaviour [17,24,62,87,131]. Thus, the presence of central sensitization in one region of the body may represent a risk factor predisposing to a more generalized central sensitization associated with pain developing in other regions of the body.

While the alterations noted above result initially from an environmental insult causing nerve damage, another factor to bear in mind is the variability between individuals and groups of individuals of different racial backgrounds in the occurrence of chronic pain, in the expression of its features, and in response to treatment (see Section 2.1 above). This suggests that genetic factors, in addition to environmental influences, may be involved in determining the expression of chronic pain, and has underpinned the recent focus in the pain field on the role of specific genes and the contribution of genetic as well as environmental factors to chronic pain. There is now some evidence that genetic factors contribute to or supplement the complex array of cellular and behavioural consequences of trigeminal nerve injury. The following will focus on this evidence, in particular that obtained from animal models of neuropathic pain over especially the past two decades, and consider the influence of genetic factors in addition to environmental factors on orofacial neuropathic pain.

## 3. Genetic and Related Environmental Factors

Studies of some chronic orofacial pain states have shown that genetic factors may contribute to pain expression or its predisposition, as well exemplified in the series of OPPERA studies that have included assessment of genetic risk factors in TMDs (e.g., [5,14,15,16]). Moreover, many pain-related or analgesia-related genes have been identified over recent decades and implicated in several pain states, including TMDs and trigeminal neuropathic pain conditions, or in response to treatment (e.g., [58,132,133,134]; for review, see [15,16,28]). Some of the identified pain-related genes are thought to be involved in determining the actual expression of pain or its level, for example following injury, while others are thought to contribute to predisposing the individual to its expression. These observations bear on the possibility that knowledge of the genetic makeup of an individual suffering from a chronic pain state may guide personalized management of the patient or help prevent the development of chronic pain in those individuals at high genetic and environmental risks for developing chronic pain. While some have questioned this view by casting doubt on whether genetic insights from investigations in humans, as well as correlated studies in rodents, can explain enough trait variance in a pain condition or in response to pain therapy to guide pain care customised for the individual chronic pain patient, many authors have enthusiastically embraced the view (for review, see [28,48,69,76,135,136]). Despite these differing views about the clinical utility of knowledge of a person’s genetic makeup, it is clear that crucial influences relevant to many models of trigeminal neuropathic pain and other chronic orofacial pain conditions are not only environmental factors but also factors that may be inherited and influence pain expression or predispose the individual to the development or maintenance of a chronic pain state. These influences were briefly mentioned above in Section 2.3, and now will be considered in more detail in relation to chronic neuropathic pain.

It has been shown that chronic neuropathic pain is a heritable, complex trait in all mammalian species studied to date, including mice, rats and humans, and that a considerable proportion of the variability in both the occurrence and level of chronic neuropathic pain after nerve injury is controlled by genetic determinants, with the contribution of heritability being estimated as moderate to high [15,28,48]. Nonetheless, while the heritability of pain traits has been well documented, environmental factors have been shown to interact with trait-relevant genes. Thus, chronic pain in the orofacial region and other parts of the body related to nerve injury is a prototypical example of gene X environment interactions through the involvement of epigenetic processes.

The importance of environmental factors was briefly noted earlier where it was pointed out that an environmental factor in the form of an injury to a nerve is the inciting event that precipitates the cascade of cellular changes leading to a chronic neuropathic pain state. However, it is also noteworthy that other environmental factors (in the broad sense of these words) may modify nociceptive behaviour and the processes underlying chronic orofacial pain. The expression of an individual’s background can be temporally and dynamically affected by environmental influences and result in intermediate phenotypes or endotypes relevant to chronic pain or its comorbidities (e.g., pain amplification, sleep disturbance, psychological distress) [3,7,9,11,28,137]. This is evident not only from observations in humans where societal factors (e.g., living environment, social context, stress, unhealthy lifestyle) as well as environmental toxins and allergens can modify pain expression and contribute to its variability between individuals, but also from studies using laboratory animal models to assess pain behaviour (e.g., [48,138,139,140,141,142]). For example, the nociceptive behaviour of a rodent may be influenced by factors such as the animal’s diet, its social interactions with littermates and its stress levels, as well as by the time of the day of the assessment of its behaviour, and even by the sex and other features of its littermates and the investigator assessing its nociceptive behaviour. Indeed, an outcome of the recent focus on pain genetics and environmental factors has been findings showing the involvement of epigenetic influences on the development and maintenance of chronic pain, and the following briefly outlines processes involved in their actions.

Epigenetic influences have been shown to be crucial for a balanced expression between pro-nociceptive genes and anti-nociceptive genes in neurons and other cellular elements involved in nociceptive transmission and its modulation. There is evidence suggesting that epigenetic influences operating through DNA methylation, noncoding RNA and chromatin remodeling may regulate the expression of genes involved in peripheral and central sensitization in humans and several animal models of pain, including neuropathic pain (for review, see [50,143,144,145]). Another related and important development has been evidence from these studies of abnormal histone acetylation and DNA methylation in glia as well as neurons, leading to dysregulation of target genes and the production of a chronic pain state, as well as the involvement in the epigenetic regulation of genes of antisense RNA and microRNA in peripheral or central nociceptive pathways. Such findings have underpinned related pre-clinical studies of the potential analgesic efficacy of epigenetic-modifying drugs. This is a particularly promising line of research in animal models of neuropathic and other types of chronic pain since it has been found that pharmacological manipulation of some of these processes (e.g., with a histone deacetylase [HDAC] inhibitor or DNA methyltransferase inhibitor) may prevent or reverse nociceptive behaviour in these models. However, the effects of such approaches are not straightforward for producing analgesia. For example, approaches that cause enhanced histone acetylation may prove to be analgesic in one chronic pain condition yet facilitate nociceptive responses in some other chronic pain condition, and administration of a HDAC inhibitor may result in upregulation of one set of genes in the CNS, yet produce downregulation of another set of genes (for review, see [143,144]). Thus, more research is needed to clarify whether and how epigenetic modifying drugs may be helpful clinically in limiting the production of a chronic pain state. This includes research into the role of epigenetic processes in orofacial pain and the efficacy of epigenetic modifying drugs in orofacial pain models. Up to now, little information is available, except for recent findings showing that the overexpression of several genes in the trigeminal ganglion and associated facial nociceptive behaviour following trigeminal nerve injury can be overcome by pre-emptive manipulation with HDAC inhibitors [146] and that DNA methylation is altered in the trigeminal ganglion following orofacial inflammation, resulting in abnormal expression of pro-nociceptive genes (e.g., TRPV1, TRPA1, P2X3; [147]).

The foregoing outline of the importance of epigenetic determinants of pain clearly has relevance to studies using animal models of trigeminal neuropathic pain and indeed other chronic orofacial pain conditions, and more studies are warranted. The design and conduct of these studies need to be mindful of environmental influences on pain and related behaviours and on the underlying peripheral mechanisms and CNS circuits and mediators through which they may act. However, environmental influences themselves may vary depending on the rodent strain examined, emphasizing the complex interactions of genetic and environmental influences on pain. Thus, studies using these models also need to be mindful of the heritability feature of chronic neuropathic pain and the contribution of genetic factors to the complex array of behavioural and cellular consequences of nerve injury since this is especially relevant to the animal species or strain selected to study genetic influences in chronic orofacial pain states or indeed other biomedical conditions.

In the case of the species selected for study, the mouse has traditionally been the favourite animal for genetics investigations but in recent years, advancements have been made in investigating the rat genome to the extent that the rat is now often used in such studies. Furthermore, even though inbred strains have usually been considered to manifest less phenotypic variability than outbred stains, comparisons of several different inbred strains have revealed considerable phenotypic and other differences, bringing into question the preference for inbred strains in genetic studies related to pain (e.g., [76,148,149]). The selection of the animal also needs to consider the possibility of strain differences in the cellular as well as the behavioural consequences of a nerve injury, since studies of strain differences could, for example, be important in providing insights into how variability occurs between humans of different racial backgrounds as well as helping to explain any differences in findings that might arise between studies in rodent pain models using the same environmental insult (e.g., similar type of nerve injury of a specific trigeminal nerve branch). The importance of not only the neuropathic pain model selected but also the strain used for the model is evident from numerous studies in rodent models of pain following injury to nerves or other tissues involving the spinal nociceptive system (e.g., [139,150,151,152,153,154,155,156,157,158,159,160,161,162,163,164,165,166]). While some differences in behavioural and cellular features have been described between some of these studies using different neuropathic pain models, significant phenotypic differences between strains have also been documented in studies that have used the same neuropathic pain model and that have controlled for sex and environmental factors such as assessment time of day and environmental and social context of the strains. These differences are apparent not only in many types of pain-like behaviour, but also in behavioural responses to analgesic approaches as well as in cellular features related to neuroplasticity and glioplasticity in the spinal nociceptive system. The following gives some examples.

Firstly, in terms of inherent differences in pain sensitivity, pain phenotypic differences were documented between two of the mouse strains (e.g., C57Bl6; 129) used to provide the default genetic profiles upon which null mutants are derived (e.g., in pain-related gene knock-out studies) [156]. In addition, thermal pain sensitivity was shown to be elevated in female Long Evans rats and Swiss Webster mice compared to males, but decreased in female Sprague–Dawley rats compared to males, and no sex differences were noted in Wistar Kyoto rats, CD-1 or ND4 mice [167]. Secondly, in models of neuropathic pain following spinal nerve injury, it was shown several years ago that two unique selectively bred strains of Sabra 2 rats expressed contrasting mechanical allodynia and heat hyperalgesia, as did WKY rats compared to several other rat strains including FIS344, SAB, SD, FSL, LEW and BN (e.g., [150,157]). Furthermore, another more recent study using different strains of rats (inbred: LEW, WKY, F344/ICO and F344/DU, outbred: Crl:SD) receiving a similar spinal nerve injury revealed significant variability between the strains in the development of mechanical allodynia and also interestingly in measures of stress, anxiety and depression which correlated only modestly with degree of pain sensitivity or with genetic predisposition to stress and/or affective disturbances [166]. Moreover, some studies have shown variability between strains not only in nociceptive behaviour following spinal nerve injury but also in markers of glioplasticity and neuroplasticity (e.g., [158,160,163,164]).

The strain differences in the expression of neuropathic pain and underlying cellular mechanisms in spinal neuropathic pain models emphasize the important contribution of genetic influences in neuropathic pain states. However, given that the trigeminal somatosensory system has several features that are different from those in the spinal somatosensory system (see Section 2.3 above), it cannot be automatically assumed that genetic factors identified in spinal neuropathic pain studies will be the same for the trigeminal system. For example, even if the same genes were found to encode the same process involved in chronic pain in both trigeminal and spinal systems, the expression levels of such genes, both constitutively and chronically, could possibly be different and change over time at a different rate. Therefore, testing for possible strain differences in genetic (as well as environmental) determinants of trigeminal neuropathic pain necessitates dedicated trigeminal models rather than relying on findings from models based on pain in other parts of the body innervated by spinal nerves. The extent to which genetically determined alterations reflected in strain differences in glioplasticity and neuronal plasticity and associated pain-related behaviour occur following nerve injury in the trigeminal system is relatively unexplored, but there have been a small number of studies testing for possible strain differences in chronic trigeminal neuropathic pain models, and they will now be considered.

The studies that have explored the possibility of strain differences in trigeminal neuropathic pain models controlled for potentially confounding factors (e.g., sex, assessment time of day, environmental and social conditions of the strains) and have revealed considerable strain variability in orofacial pain-like behaviour and in the underlying mechanism of trigeminal central sensitization. In one series of studies that included over 20 different strains of mice [118,119], variations between different strains (including A/J, C57BL/6J, DBA/2J and BXA13/PgnJ) were described not only in the expression of orofacial nociceptive behaviour but also in ETSS that lasted for several days following unilateral injury to branches of the ION (which is part of the maxillary nerve). In contrast to sham animals, mechanical and heat hypersensitivities in the ipsilateral facial region and beyond were induced by the ION injury in some but not all strains. For instance, the BXA13/PgnJ strain compared to other strains showed significantly greater heat hypersensitivity bilaterally on the ears (supplied by the mandibular nerve), paws and tail (supplied by spinal nerves), and A/J and C57BL/6J lines contrasted on heat sensitivity in the ear. Likewise, another study using this ION injury model but in rats of two different strains (WKY, LEW) showed strain differences in the magnitude and time course of hypersensitivity and ETSS in the ipsilateral face, contralateral face, and hindpaw [120], as illustrated in Figure 4 (see above). These findings in mice and rats of bilateral facial hypersensitivity following unilateral trigeminal nerve injury are consistent with several previous studies of ETSS in the face that were mentioned earlier, but the findings additionally included documentation that ETSS also occurs in widespread body regions and that the facial hypersensitivity and ETSS are strain-dependent. 

**Figure 6:**
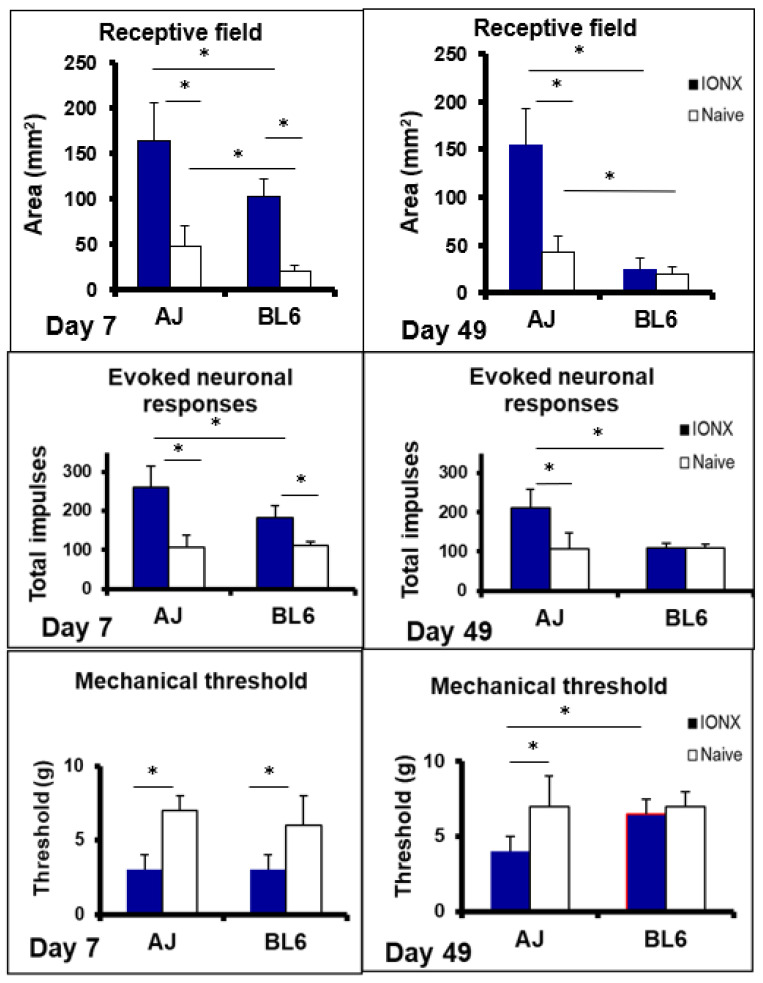
Infraorbital nerve injury in the mouse induces trigeminal central sensitization that is genetically dependent. A/J (AJ, *n* = 12) and C57BL/6J (BL6, *n* = 12) male mice aged 8–12 weeks were maintained under similar environmental conditions, and used for electrophysiological recordings made in histologically identified sites in the medullary dorsal horn. A total of 40 nociceptive neurons functionally classified as nociceptive-specific neurons was recorded. At 7 days or 49 days prior to the recording experiment, animals received unilateral transection (IONX) of the medial branch of the infraorbital nerve or no IONX (i.e., naïve rats). IONX-induced trigeminal central sensitization of the neurons was expressed as significant increases (compared to naïve) in neuronal mechanoreceptive field size and responses evoked by graded mechanical stimulation and a significantly decreased mechanical activation threshold (ANOVA, *p* < 0.05). Note the significant differences between A/J and C57BL6 mice in the magnitude and time course of these three parameters of trigeminal central sensitization following IONX (2-way ANOVA, *p* < 0.05). Central sensitization was evident at post-operative day 5 in both A/J and C57BL6 mice but at post-operative day 49 it was only present in A/J mice. Note in addition that naïve animals also showed a significant difference between A/J and C57BL6 mice in mechanoreceptive field size. * *p* < 0.05. Based on data from Varathan et al. [121] and Cherkas et al. [168].

There are also related findings indicating that strain differences in trigeminal central sensitization may underlie the strain differences in the associated nociceptive behaviour following trigeminal nerve injury [121,168]. In this study that also used the ION injury model, A/J and C57BL/6J mice showed similar baseline values of facial mechanosensitivity, but unilateral injury of the ION produced a significant increase in mechanosensitivity reflecting nociception contralaterally as well as ipsilaterally in both A/J and C57BL/6J mice. However, as illustrated in Figure 5, the time course of the mechanical hypersensitivity was significantly different between the two strains of mice. Moreover, strain differences were also found in the immunohistochemically labelling of astrocytes and microglia examined in the MDH at post-operative days 5 and 28 following the ION injury. While both A/J and C57BL6 mice showed significantly enhanced labelling in the ipsilateral and contralateral MDH of astrocytes (labelled with GFAP) and microglia (labelled with Iba1) following IONX (2-way ANOVA, *p* < 0.05), the labelling at post-operative day 5 was 60–70% greater in the A/J mice in which the increased labelling was still present at post-operative day 28; as Figure 5 shows, the nociceptive behaviour was also significantly greater in the A/J mice at these two time points. Furthermore, these differences between the two strains in nociceptive behaviour and in the immunohistochemical expression of glioplasticity in the MDH were complemented by findings of electrophysiologically defined neuroplasticity reflecting trigeminal central sensitization in the MDH. Electrophysiological recordings made in the MDH of these two strains of mice revealed that ION injury induced trigeminal central sensitization that was different in magnitude and duration depending on the strain of the animal. Central sensitization (manifested as increases in MDH neuronal receptive field size and responses to orofacial stimuli and reduced mechanical activation threshold) was evident in both strains of mice at post-operative day 7, but its magnitude was significantly greater in the A/J strain at this time point (Figure 6). The duration of central sensitization was also significantly longer in the A/J mice since its presence still at post-operative day 49 in the A/J mice contrasted with its absence in the C57BL6 mice at this time point. It also closely matched the time course of the nociceptive behaviour which had reached its peak by post-operative day 7 and also lasted up to post-operative day 49 following the ION injury in the A/J strain, whereas the nociceptive behaviour had already returned to baseline levels by post-operative day 28 in the C57BL6 strain (Figure 5).

Collectively, the studies outlined above using rodent models of trigeminal neuropathic pain have revealed that the predisposition to nerve injury-induced nociceptive behaviour as well as to MDH glioplasticity and neuroplasticity underlying trigeminal central sensitization varies markedly between genetically different rodent strains. The findings of strain differences indicate that trigeminal central sensitization and the associated expression of features of nociceptive behaviour such as hypersensitivity and ETSS following trigeminal nerve injury may be under genetic control. The findings are consistent with earlier evidence of strain differences in spinal neuropathic pain models, and suggest that genetic factors influencing central sensitization may contribute to the variation between clinical cases in the development, expression and maintenance of trigeminal neuropathic pain and other chronic orofacial pain conditions in humans. Nonetheless, in the case of trigeminal neuropathic pain, it is important to be mindful that the strain-dependent effects of trigeminal nerve injury may extend beyond the trigeminal nociceptive system. It has been well-documented that ION injury or other manipulations of orofacial tissues can produce neuroplastic changes that occur in several CNS areas that are components of the rodent trigeminal somatosensory or motor systems (for review, see [24,169,170,171,172]) and that indeed may encompass other CNS areas involved in cognitive, memory and affective functions depending on the strain of the rodent model being investigated (e.g., [169]). Such findings underscore the need for more investigations into the role of genetic factors in the expression of not only pain but also in related behaviours in animal models involving orofacial injury. The insights gained from these investigations will provide a more comprehensive picture of the strain-dependent effects and underlying mechanisms that will be useful in informing the management of trigeminal neuropathic pain and associated conditions.

## 4. Conclusions

This review has outlined features of chronic orofacial pain states in humans as well as findings from animal models of the behavioural characteristics and underlying mechanisms related to the development, expression and maintenance of chronic orofacial pain and trigeminal neuropathic pain in particular. The role of environmental and especially genetic factors in these models has also been reviewed, and findings described of differences between strains of animals in the features and some of the mechanisms involved in chronic pain. These findings have revealed that the predisposition to nerve injury-induced nociceptive behaviour as well as to trigeminal central sensitization may show considerable variability between genetically different rodent strains. The findings are relevant to clarifying the mechanisms underlying the variability between patients in the features of chronic orofacial pain, and also underscore the careful consideration needed in the selection of the strain(s) of the animal for those studies planning to model a chronic orofacial pain condition and explore underlying processes.

## Data Availability

Not applicable.

## Abbreviations

ATP	adenosine tri-phosphate
BMS	burning mouth syndrome
CB	cannabinoid
CGRP	calcitonin gene related peptide
CNS	central nervous system
CUPFA	Constant Unilateral Facial Pain with Additional Attacks
ETSS	Extra-territorial spread of sensitivity
IAN	inferior alveolar nerve
ION	infraorbital nerve
MDH	medullary dorsal horn
NGF	nerve growth factor
NMDA	N-Methyl-D- aspartate
OPPERA	Orofacial Pain Prospective Evaluation and Risk Assessment
p-ERK	phosphorylated extracellular signal-regulated kinase
PIDP	persistent idiopathic dentoalveolar pain
PIFP	persistent idiopathic facial pain
PTNP	post-traumatic trigeminal neuropathic pain
P2X	purinergic receptor 2X
P2Y	purinergic receptor 2Y
TBSNC	trigeminal brainstem sensory nuclear complex
TMDs	temporomandibular disorders
TNF	Tumor necrosis factor
TRPV1	Transient Receptor Potential Vanilloid 1
